# Performance of the SCORE and Globorisk cardiovascular risk prediction models: a prospective cohort study in Dutch general practice

**DOI:** 10.3399/BJGP.2021.0726

**Published:** 2022-11-29

**Authors:** Merle CA Schoofs, Reinier P Akkermans, Wim JC de Grauw, Bianca WM Schalk, Ineke van Dis, Judith Tjin-A-Ton, Erik WMA Bischoff, Marion CJ Biermans

**Affiliations:** Department of Primary and Community Care, Radboud University Medical Center, Nijmegen, the Netherlands.; Department of Primary and Community Care and Department of IQ healthcare, Radboud Institute for Health Sciences, Radboud University Medical Center, Nijmegen, the Netherlands.; Department of Primary and Community Care, Radboud University Medical Center, Nijmegen, the Netherlands.; Department of Primary and Community Care, Radboud University Medical Center, Nijmegen, the Netherlands.; Dutch Heart Foundation, The Hague, the Netherlands.; GP practice Frakking & Tjin- A-Ton, Amstelveen, the Netherlands.; Department of Primary and Community Care, Radboud University Medical Center, Nijmegen, the Netherlands; Department of Primary and Community Care, Radboud University Medical Center, Nijmegen, the Netherlands.

**Keywords:** cardiovascular disease, prospective studies, primary health care, risk equation

## Abstract

**Background:**

GPs frequently use 10-year-risk estimations of cardiovascular disease (CVD) to identify high- risk patients.

**Aim:**

To assess the performance of four models for predicting the 10-year risk of CVD in Dutch general practice.

**Design and setting:**

Prospective cohort study. Routine data (2009– 2019) was used from 46 Dutch general practices linked to cause of death statistics.

**Method:**

The outcome measures were fatal CVD for SCORE and first diagnosis of fatal or non- fatal CVD for SCORE fatal and non-fatal (SCORE- FNF), Globorisk-laboratory, and Globorisk-office. Model performance was assessed by examining discrimination and calibration.

**Results:**

The final number of patients for risk prediction was 1981 for SCORE and SCORE-FNF, 3588 for Globorisk-laboratory, and 4399 for Globorisk- office. The observed percentage of events was 18.6% (*n* = 353) for SCORE- FNF, 6.9% (*n* = 230) for Globorisk-laboratory, 7.9% (*n* = 323) for Globorisk-office, and 0.3% (*n* = 5) for SCORE. The models showed poor discrimination and calibration. The performance of SCORE could not be examined because of the limited number of fatal CVD events. SCORE-FNF, the model that is currently used for risk prediction of fatal plus non-fatal CVD in Dutch general practice, was found to underestimate the risk in all deciles of predicted risks.

**Conclusion:**

Wide eligibility criteria and a broad outcome measure contribute to the model applicability in daily practice. The restriction to fatal CVD outcomes of SCORE renders it less usable in routine Dutch general practice. The models seriously underestimate the 10-year risk of fatal plus non-fatal CVD in Dutch general practice. The poor model performance is possibly because of differences between patients that are eligible for risk prediction and the population that was used for model development. In addition, selection of higher-risk patients for CVD risk assessment by GPs may also contribute to the poor model performance.

## INTRODUCTION

Cardiovascular disease (CVD) is the leading cause of hospital admissions and deaths worldwide, contributing to over 30% of global deaths.^1^^,^^2^ Treatment based on 10-year-risk estimations of CVD is included in many clinical guidelines for primary prevention of CVD.^3^^–^^5^ GPs play a key role in the prevention of CVD. They use the 10-year-risk estimations to identify patients with a high risk for CVD and to communicate this risk to their patients. After explaining the potential risks and benefits of treatment, they decide with their patients which preventive action will be taken, such as lifestyle interventions or prescribing medication. For this informed shared decision making, it is essential that risk prediction models are accurate.

In the past decades, numerous models that predict 10-year risk of CVD have been developed.^6^ Two commonly known models are SCORE^7^ and Globorisk.^8^ Globorisk has two versions: one based on laboratory measurements (Globorisk-laboratory, henceforth referred to as Globo-lab) and one based on office measurements only, which are measurements that do not require blood tests (Globorisk-office, henceforth referred to as Globo-office).^9^

In the Netherlands, the guideline on cardiovascular risk management (CVRM) advises GPs to regularly measure all cardiovascular risk factors and assess 10-year risk of CVD in high-risk patients, including patients with a family history of CVD or dyslipidaemia, risk factors such as smoking, obesity, elevated blood pressure or cholesterol levels, and comorbidities such as diabetes mellitus, rheumatoid arthritis, or chronic obstructive pulmonary disease. SCORE for low-risk countries (that is, a SCORE version for low-risk European regions based on mortality statistics) is adopted in this guideline.^10^ SCORE predicts the 10-year risk of fatal CVD. In addition, the Dutch guideline reports the 10-year risk of fatal and non-fatal CVD, which is called SCORE fatal and non-fatal (SCORE-FNF) throughout this article, where the SCORE risk for low-risk countries is multiplied by coefficients based on data from the Dutch EPIC-NL cohort.^10^^–^^12^ The derivation cohorts of SCORE, SCORE-FNF, and Globorisk are described in Supplementary Tables S1–S3.

Although SCORE and SCORE-FNF are applied in Dutch general practice, they have not been evaluated in this setting. Before a predictive model can be used in a different setting than the one from which it was derived, it should be successfully externally validated in this new setting. In such a domain validation, the potential for differences between the derivation and validation population is large.^13^

**Table:** How this fits in

Many prediction models estimating the risk of cardiovascular disease (CVD), such as SCORE and Globorisk, have been developed in cohorts from a general population. Although SCORE is applied in Dutch general practice, it has not been evaluated in this setting. Also, an external validation of the Globorisk models in a Dutch general practice setting is lacking. This study found that SCORE fatal and non- fatal (SCORE- FNF), Globorisk-laboratory, and Globorisk-office underestimate the 10-year risk of fatal plus non-fatal CVD in most of the patients who have their risk assessed by GPs. This study also showed that outcome definitions and eligibility criteria differed among the models, influencing their clinical applicability.

SCORE is evaluated in population-based studies in various countries,^6^^,^^14^^,^^15^ including several samples from the general Dutch population.^16^^–^^19^ Although some SCORE studies recruited patients from general practice,^20^^–^^24^ no study evaluated the real-life use of SCORE by GPs in patients selected by the GP for CVRM. Globorisk has been evaluated in some population-based studies,^14^^,^^25^^,^^26^ but not in general practice.

The aim of this study was to validate SCORE, SCORE- FNF, Globo-lab, and Globo-office using real- life data from Dutch general practices for patients who were selected for risk assessment by GPs. The applicability and performance of these four models were examined.

## METHOD

### Design, setting, and selection study population

This study prospectively reviewed data from electronic health records in the general practice database of the Department of Primary and Community Care at the Radboud University Medical Center. Practices (*n* = 46) were selected that provided data at baseline, that is, 1 January 2009. All practices provided data for at least 10 years. Patients were included if they were registered at one of these practices at baseline. For the evaluation of SCORE and SCORE-FNF, patients aged 40–70 years without a history of diabetes and CVD at baseline were included.^10^ For the evaluation of Globo-lab and Globo- office, patients aged 40–74 years without a history of stroke or coronary heart disease were included. Patients were included if predictors were measured by GPs between 1 July 2008 and 1 January 2009. This 6-month period was chosen because it is relatively close to baseline and therefore provides a reasonable reliable estimation of the true value of the predictors at baseline. The authors of the current study assumed that patients with these predictor measurements (see Supplementary Table S4) were selected for risk assessment by GPs, which is a valid assumption according to the GPs in the current study’s research team. To determine the smoking status of the patients, data from between 1 January 2008 and 1 January 2019 was used (see Supplementary Box S1 for more details). Patients were included based on the inclusion and exclusion criteria of the prediction models (see Supplementary Table S4), resulting in three study populations. Each participant could be included in more than one study population.

### Prediction models

The SCORE and SCORE-FNF models are based on age, sex, systolic blood pressure (SBP), smoking status, total cholesterol/high-density lipoprotein cholesterol ratio, and rheumatoid arthritis.^7^^,^^10^ To calculate the risk of fatal plus non-fatal CVD based on SCORE-FNF, the risks calculated based on SCORE were multiplied by age- and sex- specific multipliers (see Supplementary Box S2).^10^

Globo-lab uses information on age, sex, SBP, diabetes, smoking status, and total cholesterol. In the Globo-office model, body mass index replaces total cholesterol and diabetes. These Globorisk models were recalibrated using age- and sex-specific mean risk factor levels and CVD rates for the Netherlands.^9^

### CVD outcomes

The CVD outcomes of the models are as follows:

- SCORE predicts risk of fatal CVD with an atherosclerotic cause, including ischaemic heart disease, stroke, and abdominal aorta aneurysm;
- SCORE-FNF predicts risk of fatal CVD with an atherosclerotic cause plus hospital admission for myocardial infarction, heart failure, stroke, and peripheral vascular disease; and
- Globo-lab and Globo-office predict risk on fatal ischaemic heart disease, stroke, or sudden cardiac death, and non-fatal myocardial infarction or stroke (International Classification of Diseases, 10th Revision [ICD-10] codes are presented in Supplementary Table S5).

All non-fatal outcomes were converted into international classification of primary care (ICPC)-1 and ICPC-2 codes, as diagnoses were coded according to the ICPC-1, ICPC-2, or ICD-10 in the present authors’ database.^27^^,^^28^ To determine the cause and date of death of patients who died within the study period, general practice data were linked to cause of death statistics (ICD-10 codes) from Statistics Netherlands based on sex, date of birth, and four-digit postal code.

### Statistical analyses

To assess the applicability of the prediction models, the final number of patients and events for each model were assessed. Discrimination was assessed by calculating Harrell’s *C* statistic.^29^^–^^31^ Calibration was visually assessed using calibration plots. Predicted risks were plotted against the observed risks, where patients were grouped by decile of predicted risk. The observed CVD risk was obtained using 10-year Kaplan– Meier estimates.^32^ In addition, flexible calibration curves were plotted using three-knot restricted cubic splines.^33^ Also the Integrated Calibration Index (ICI), E50, and E90 were calculated (see Supplementary Box S3 for more details).

Examination of the performance of SCORE was not possible because of the low number of fatal CVD events (*n* = 5). Comparison with Statistics Netherlands CVD mortality data and the declining trend in Dutch CVD mortality rates revealed that this was a plausible number of fatal CVD events.^34^^–^^36^

Several analyses were performed to get more insight into the selection of patients for risk assessment by GPs (see Supplementary Box S4 for more details). Statistical analyses were performed using IBM SPSS Statistics (version 25) or R (version 3.6.3).

## RESULTS

### Study populations

**Figure 1.**
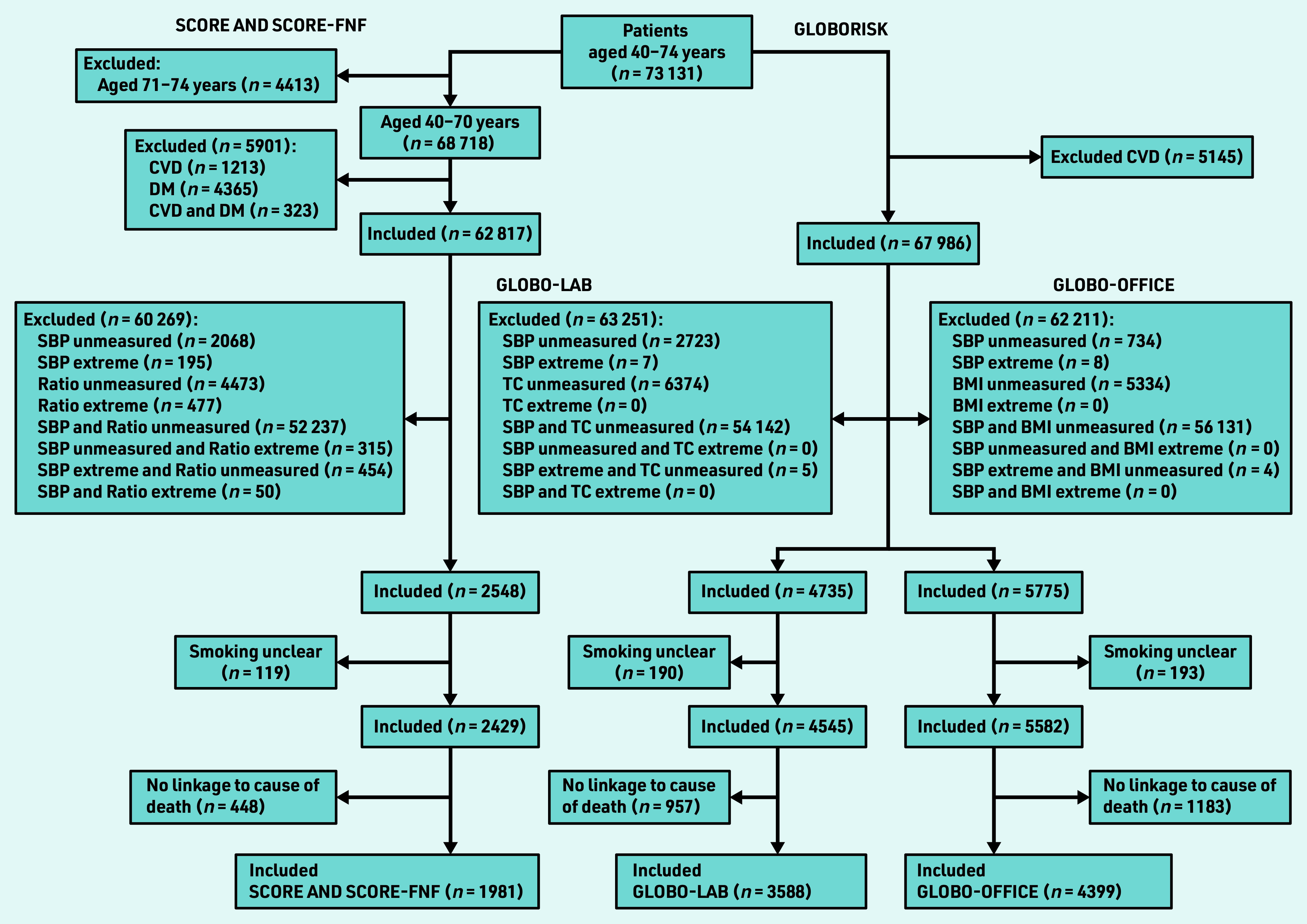
*Flowchart of the selection of the study population. BMI = body mass index. CVD = cardiovascular disease. DM = diabetes mellitus. Globo-lab = Globorisk-laboratory. Globo- office = Globorisk-office. Ratio = total cholesterol/high-density lipoprotein cholesterol ratio. SBP = systolic blood pressure. SCORE-FNF = SCORE fatal and non-fatal. TC = total cholesterol.*

Figure 1 presents a flowchart of the study population selection. In total, 1981 patients were included in the SCORE populations, 3588 in the Globo-lab population, and 4399 in the Globo-office population.

Supplementary Table S6 presents characteristics of patients that could and could not be linked to cause of death statistics. The mean follow-up time was 8.4 (SD 2.9) years for SCORE and 9.0 (SD 2.3) years for Globo-lab and Globo- office. Table 1 presents baseline values on predictors.

**Table 1.** Characteristics of included patients at baseline^a^

**Characteristic**	**SCORE/SCORE-FNF**	**Globo-lab**	**Globo-office**
*n*	1981	3588	4399
Age, years, mean (SD)	57.4 (7.7)	58.9 (8.6)	59.7 (8.6)
Female, *n* (%)	1026 (51.8)	2018 (56.2)	2412 (54.8)
SBP, mmHg, mean (SD)	142 (14)	141 (17)	140 (16)
TC, mmol/L, mean (SD)		5.4 (1.2)	
TC/HDL ratio, mean (SD)	4.6 (1.1)		
Diabetes, *n* (%)	0 (0.0)	1045 (29.1)	
Rheumatoid arthritis, *n* (%)	26 (1.3)	—	—
Smoker, *n* (%)	239 (12.1)	502 (14.0)	700 (15.9)
BMI, kg/m^2^, mean (SD)	—	—	29.1 (5.1)

a *^—^ = predictor is not included in the model. BMI = body mass index. Globo-lab = Globorisk-laboratory. Globo-office = Globorisk-office. HDL = high-density lipoprotein. SBP = systolic blood pressure. SCORE-FNF = SCORE fatal and non-fatal. SD = standard deviation. TC = total cholesterol.*

### Applicability

The 10-year observed risks are 18.6% (*n* = 353) for SCORE-FNF, 6.9% (*n* = 230) for Globo-lab, and 7.9% (*n* = 323) for Globo- office population (see Supplementary Table S7). Of these events, only three additional events for SCORE and four for Globo-lab and Globo- office were found by linkage to cause of death data from Statistics Netherlands (see Supplementary Table S8).

### Performance of prediction models

**Figure 2.**
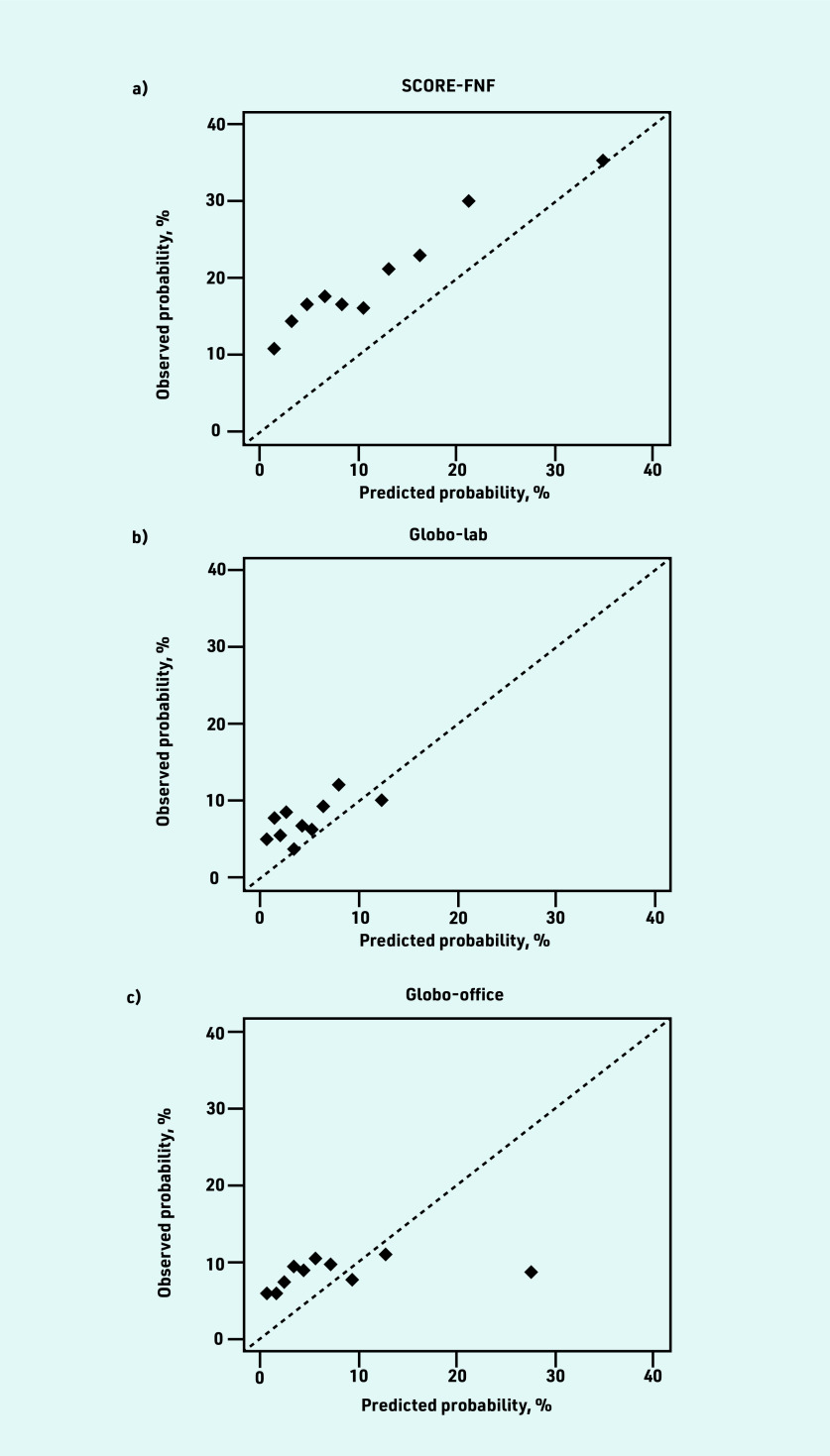
*Calibration plots of predicted versus observed risks of a) SCORE-FNF; b) Globo- lab; and c) Globo- office. Patients were  grouped based on deciles of predicted risk. The diagonal line represents the line of perfect calibration. See Supplementary Table S7 for more details. Globo- lab = Globorisk- laboratory.  Globo- office = Globorisk-office. SCORE-FNF = SCORE  fatal and non-fatal.*

**Figure 3.**
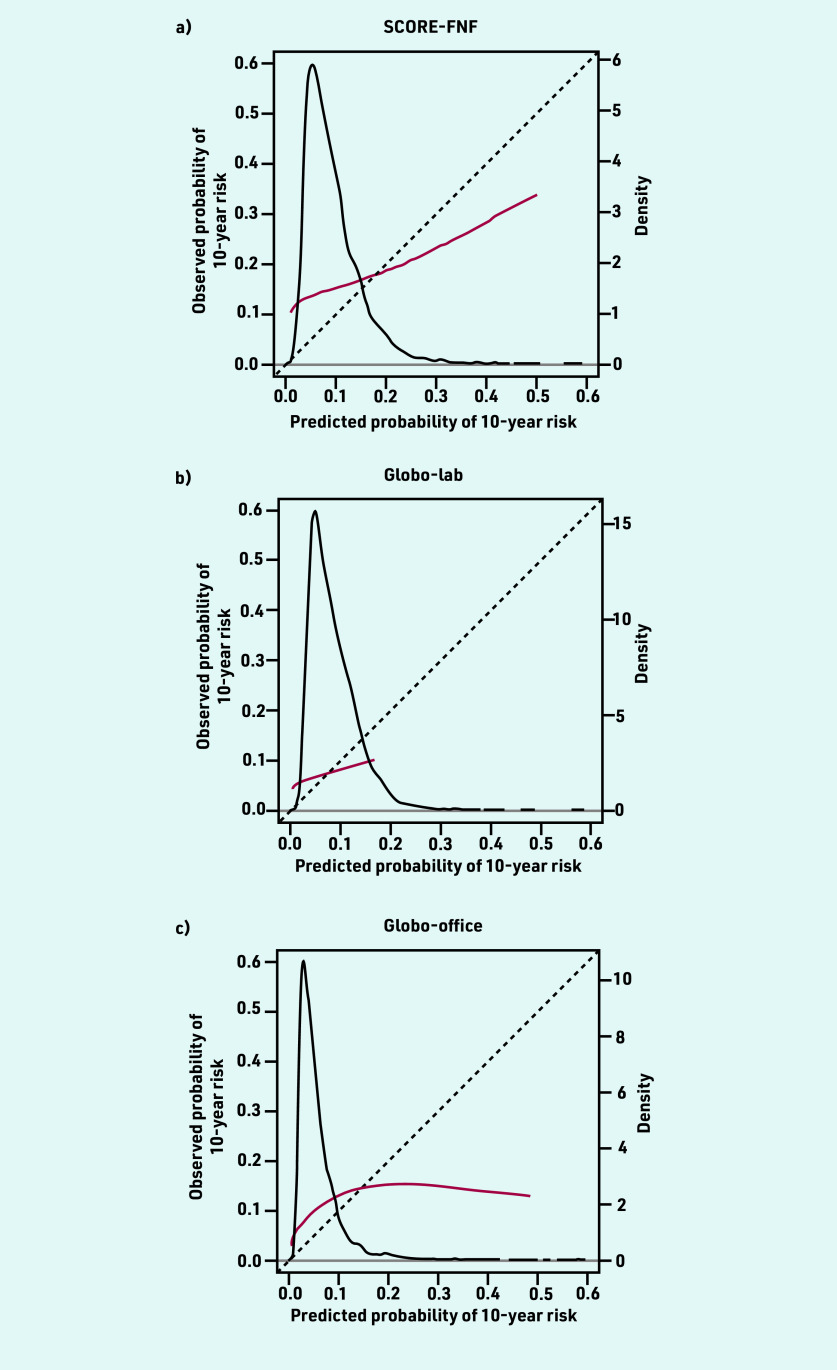
*Calibration plots of predicted versus observed risks of a) SCORE-FNF; b) Globo-lab; and c) Globo-office using restricted cubic splines. The red lines denote the calibration curves. The diagonal line represents the line of perfect calibration. The density function (black line) shows the distribution of predicted risk (right y-axis). Globo-lab = Globorisk-laboratory. Globo-office = Globorisk-office. SCORE-FNF = SCORE non-fatal and fatal.*

The mean predicted CVD risk was 12.2% for SCORE-FNF, 4.8% for Globo-lab, and 7.8% for Globo-office. The *C* statistic was 0.613 (95% CI = 0.579 to 0.646) for SCORE- FNF, 0.561 (95% CI = 0.522 to 0.600) for Globo- lab, and 0.539 (95% CI = 0.508 to 0.570) for Globo-office. The ICI was 6.9% for SCORE-FNF, 8.8% for Globo-lab, and 8.4% for Globo-office (Table 2). The calibration plots of the three models showed an underestimation in almost all deciles of predicted risk (Figure 2). The models underestimated the risk for patients with lower predicted risks and overestimated the risk for patients with higher predicted risks, but higher predicted risks were less frequent (Figure 3).

**Table 2.** Observed and predicted 10-year risk of cardiovascular events by Globo-lab, Globo-office, and SCORE-FNF^a^

**Measure**	**SCORE-FNF**	**Globo-lab**	**Globo-office**
*C* statistic (95% CI)	0.613 (0.579 to 0.646)	0.561 (0.522 to 0.600)	0.539 (0.508 to 0.570)
Predicted events, %	12.2	4.8	7.8
ICI, %	6.9	8.8	8.4
E50, %	7.4	8.9	8.6
E90, %	8.5	9.4	9.3

a *See Supplementary Box S3 for more details of ICI, E50, and E90. ICI = Integrated Calibration Index. Globo-lab = Globorisk-laboratory. Globo-office = Globorisk-office. SCORE-FNF = SCORE fatal and non-fatal.*

### Selection of patients

No large differences were found in age and sex distribution between the initial general practice population (*n* = 73 131) and the general Dutch population (see Supplementary Figure S1). Figure 1 shows that GPs measured predictors only in a limited part of the population. More predictor measurements were performed in older patients and in females aged ≥50 years versus males aged ≥50 years (Table 3). The CVD incidence rate in the SCORE-FNF cohort was lower than in the eligible general practice population for SCORE-FNF; 4.16 and 9.57 per 1000 patients per year, respectively (see Supplementary Table S9). The CVD incidence rate in the Globorisk cohort was 8.04 compared with 7.54 per 1000 patients per year in the eligible general practice population for Globorisk (see Supplementary Table S10).

**Table 3.** Characteristics of patients that could and could not be included in the models based on availability of predictor measurements

**Characteristics**	**Included Globo-office**	**Excluded Globo-office**	**Included Globo-lab**	**Excluded Globo-lab**	**Included SCORE/SCORE-FNF**	**Excluded SCORE/SCORE-FNF**
***n***	5582	62 404	4545	63 441	2429	60 388

**Females, *n* (%)**	2993 (53.6)	32 188 (51.6)	2524 (55.5)	32 657 (51.5)	1265 (52.1)	30 964 (51.3)

**Females by age group, *n* (%)**						
40 49 years	379 (6.8)	12 903 (20.7)	354 (7.8)	12 928 (20.4)	209 (8.6)	12 920 (21.4)
50 59 years	936 (16.8)	10 462 (16.8)	851 (18.7)	10 547 (16.6)	489 (20.1)	10 599 (17.6)
60 69 years	1175 (21.0)	6802 (10.9)	944 (20.8)	7033 (11.1)	524 (21.6)	7000 (11.6)
70–75 years	503 (9.0)	2021 (3.2)	375 (8.3)	2149 (3.4)	43 (1.8)	445 (0.7)

**Males by age group, *n* (%)**						
40 49 years	403 (7.2)	12 459 (20.0)	356 (7.8)	12 506 (19.7)	234 (9.6)	12 393 (20.5)
50 59 years	832 (14.9)	10 044 (16.1)	669 (14.7)	10 207 (16.1)	417 (17.2)	10 128 (16.8)
60 69 years	970 (17.4)	6214 (10.0)	744 (16.4)	6440 (10.2)	479 (19.7)	6523 (10.8)
70–75 years	384 (6.9)	1499 (2.4)	252 (5.5)	1631 (2.6)	34 (1.4)	380 (0.6)

*Globo-lab = Globorisk-laboratory. Globo-office = Globorisk-office. SCORE-FNF = SCORE fatal and non-fatal.*

The baseline age and SBP in the source population of the SCORE-FNF cohort, in which everyone was measured, was 49 (SD 12) years and 126 (SD 19) mmHg. In the current general practice source population, in which a selection of patients were measured, the baseline age and SBP were 53 (SD 11) years and 142 (SD 16) mmHg (see Supplementary Table S11).

## DISCUSSION

### Summary

SCORE-FNF, Globo-lab, and Globo-office underestimate the 10-year risk of fatal plus non-fatal CVD in most of the patients who were selected for risk assessment by GPs. Because of the low number of CVD deaths (*n* = 5) in the SCORE population, it was not possible to assess the performance of SCORE, which predicts 10-year risk of fatal CVD only. This raises questions regarding the applicability of SCORE as only 0.3% of the SCORE population had a fatal CVD event with the pre-specified ICD-10 codes. Considering the final number of patients, the Globorisk models with 3588 and 4399 patients were more widely applicable than the SCORE models with 1981 patients.

The difference in the CVD incidence rate between the EPIC-NL and general practice cohort (see Supplementary Table S9) indicates that the miscalibration of SCORE- FNF is partly because of differences in incidence between the derivation and validation population.

### Strengths and limitations

Major strengths of the present study are that the data were linked to cause of death statistics from Statistics Netherlands and the analysis was performed in general practice patients with a 10-year follow-up period, which is a representative population for the real-life setting where CVD risk charts are used. This study also has some limitations that require consideration.

First, medication use before and during the 10-year follow-up period for patients was not taken into account. Patients at increased risk of CVD may have taken medication to lower their CVD risk, which may have influenced the results. Second, general practice data on CVD diagnoses were used instead of data on CVD hospital admissions. In the Netherlands, GPs receive a letter with the diagnoses of their patients after admission to hospital. These diagnoses, especially such serious diagnoses as CVDs, are usually carefully recorded by the GPs, and will therefore be included in the present data. However, the authors might have overestimated the number of CVD events because not all patients with CVD in primary care are referred to the hospital and some CVDs might be incorrectly diagnosed in primary care. This applies, in particular, to peripheral artery disease and heart failure.^37^^,^^38^ However, based on the Dutch clinical guideline^39^ and clinical expertise in the research team, the authors do not expect that these missed diagnoses or possible outcome misclassification had a large impact on the present results as peripheral artery disease and heart failure occur mostly in older individuals, who were not included in this study.^40^^,^^41^

Third, the assumption that patients with complete measurements on all predictor variables are the patients who were selected by GPs for cardiovascular risk assessment may not hold for patients in the Globo-office population who did not have a cholesterol measurement. Fourth, the 2006 risk assessment standard,^42^ which was applicable at baseline in 2009, changed in 2011^43^ and 2019.^10^ The patient population that was selected for risk assessment changed over time (especially the diagnoses rheumatoid arthritis and chronic kidney disease were added as indicators for risk assessment). The present cohort may therefore be less representative for present- day patients. Fifth, the *C* statistic^29^^–^^31^ in the current study may be biased as it assumes that censoring is random. It is unlikely that the censoring is random, because patients at risk of CVD are likely to be at higher risk of non-CVD death. However, only 1.4%– 2.5% of the population studied died from a non- CVD cause (see Supplementary Table S8). The authors therefore believe that this bias is relatively small.

### Comparison with existing literature

The selection of patients may explain why SCORE-FNF, Globo-lab, and Globo- office underestimate the risk of fatal plus non- fatal CVD in the Dutch general practice setting. The models are mostly developed with data from the general population, whereas Dutch GPs apply the models mainly to patients with a suspected cardiovascular risk. This is in contrast with England, where the NHS Health Check programme involves a systematic CVD risk assessment in all individuals aged 40–74 years without existing CVD, diabetes, and other cardiometabolic diseases.^44^ Obviously, the patients screened by the Dutch GPs belong to a population with a greater rate of disease. Previous research has shown that multimorbidity was related to more GP consultations.^45^^,^^46^ The present study selected patients who had a clinical history available that included information on risk factors that are needed for risk prediction. Therefore, patients in the present study are more likely to represent a more diseased population than model development cohorts (Table 3). Glynn *et al* in 2008 showed that multimorbidity increases the risk of the development of CVD, which might have contributed to the underestimation that was found in the present study in the general practice population.^47^

The present results are in line with a study on the performance of SCORE-FNF in general practices in Norfolk in the UK, which found that the risk charts of the previous Dutch GP’s guideline on CVRM seriously underestimate the risk of non- fatal CVD.^22^ To the authors’ knowledge, no study has evaluated the performance of the Globorisk models in a general practice setting.

### Implications for research and practice

The authors suggest a re-estimation of the calibration coefficients using routine, pooled data from various general practice registries. This would improve the generalisability of the prediction model in the general practice setting.^13^

Besides re-estimation of coefficients, the addition of new predictors may also improve the performance of risk prediction models.^13^ The low model performances imply that the incorporation of new risk factors may be needed to improve risk prediction. A predictor that is gaining more attention in the field of CVD risk prediction is social deprivation, which is related to CVD risk.^48^^,^^49^ Social deprivation has been incorporated in the QRISK models that predict CVD risk.^50^^–^^52^ These models have been developed using general practice data and have been shown to perform well in a general practice population in the UK.^52^

In this study it was not possible to assess the performance of SCORE, as only five fatal CVD events were observed. Although CVD death is the most robust clinical outcome measure, it is less relevant if it occurs so seldomly in this timeframe, with these selection criteria, and these pre- specified ICD-10 codes. Considering the low applicability, it could be questioned whether SCORE should be used at all in the Dutch general practice setting. The SCORE- FNF and Globorisk models are clinically more relevant than SCORE as they include both CVD mortality and CVD morbidity as outcome measures. When compared with the Globorisk models, SCORE-FNF showed a greater applicability regarding the outcome definition. This is mainly because of the inclusion of heart failure and peripheral vascular disease in the outcome definition, which resulted in more events being observed in this population than in the Globorisk population. Another factor that contributes to the model applicability includes the eligibility criteria. The authors found in the present data that about twice as many patients could be included for risk prediction using the Globorisk models than using the SCORE models. Rather than only looking at the predictive performance of the models, the authors think that more attention should be paid to the model applicability, which covers CVD outcome definition and eligibility criteria.

In conclusion, this study found that SCORE-FNF, Globo-lab, and Globo-office underestimate the 10-year risk of fatal plus non-fatal CVD in most of the patients that were selected for risk assessment by GPs. Two reasons may contribute to this. First, differences in CVD incidence between derivation and validation population may contribute to the miscalibration of SCORE- FNF. Second, GPs probably select patients with more disease for CVD risk prediction than patients from the general population that were incorporated in the development cohorts of these models. Re-estimation of coefficients using general practice data and incorporating new risk factors in prediction models may improve risk prediction in a general practice setting. The authors believe that deciding which prediction model should be used for CVD risk prediction in general practice should not only rely on model performance but also on the applicability of the models, which includes broad eligibility criteria and a clinically relevant and frequently occurring outcome definition.

## Data

The datasets used during the present study are available from the corresponding author on reasonable request.
